# Treatment of Hereditary Angioedema: items that need to be addressed in practice parameter

**DOI:** 10.1186/1710-1492-6-11

**Published:** 2010-05-25

**Authors:** Callie Dagen, Timothy J Craig

**Affiliations:** 1 College of Medicine, Penn State University, Hershey, PA, USA; 2 Section of Allergy Asthma and Immunology, Medicine and Pediatrics, Penn State University, 500 University Drive, Hershey, PA 17033, USA

## Abstract

**Background:**

Hereditary Angioedema (HAE) is a rare, autosomal dominant (AD) disorder caused by a C1 esterase inhibitor (C1-inh) deficiency or qualitative defect. Treatment of HAE in many parts of the world fall short and certain items need to be addressed in future guidelines.

**Objective:**

To identify those individuals who should be on long-term prophylaxis for HAE. Additionally, to determine if prodromal symptoms are sensitive and specific enough to start treatment with C-1 INH and possibly other newly approved therapies. Also, to discuss who is appropriate to self-administer medications at home and to discuss training of such patients.

**Methods:**

A literature review (PubMed and Google) was performed and articles published in peer-reviewed journals, which addressed HAE prophylaxis, current HAE treatments, prodromal symptoms of HAE and self-administration of injected home medications were selected, reviewed and summarized.

**Results:**

Individuals whom have a significant decrease in QOL or have frequent or severe attacks and who fail or are intolerant to androgens should be considered for long-term prophylaxis with C1INH. Prodromal symptoms are sensitive, but non-specific, and precede acute HAE attacks in the majority of patients. Although the treatment of prodromal symptoms could lead to occasional overtreatment, it could be a viable option for those patients able to adequately predict their attacks. Finally, self-administration, has been shown to be feasible, safe and effective for patients who require IV therapy for multiple other diseases to include, but not limited to, hemophilia.

**Conclusions:**

Prophylactic therapy, treatment at the time of prodromal symptoms and self-administration at home all should allow a reduction in morbidity and mortality associated with HAE.

## Background

Hereditary angioedema (HAE) is a rare autosomal dominant disease with significant mortality and morbidity. HAE involves an absent or dysfunctional C-1 esterase inhibitor (C1-inh), which is a multifactorial protease involved in the control of vascular permeability. C1-inh is involved in the regulation of the complement, contact, coagulation and fibrinolytic systems. It is the main inhibitor of C1r and C1s of the complement system. C1-inh is also a major inhibitor of factor XII and kallikrein of the contact system, and to a lesser extent of factor XI and tissue-type plasminogen. Finally, C1-inh controls the production of vasoactive peptides, of which bradykinin has been significantly implicated in the development of angioedema [1,2].

Clinically, HAE is characterized by acute attacks of painless, non-pitting, non-pruritic swelling of the skin and subcutaneous tissues. It affects approximately 1 in 10000 to 1 in 50000 individuals of all races and ethnicities. Due to its significant morbidity and its 15-33% mortality, usually due to laryngeal edema and subsequent asphyxiation, some individuals require long term prophylaxis in order to prevent subsequent attacks [3-5]. Currently, medications used for prophylaxis largely revolve around the androgen, danazol, although prophylactic treatment with C1 esterase inhibitor is now available. Danazol, though effective in decreasing the severity and frequency of attacks, has numerous side effects, which often leads to its discontinuation or patient noncompliance [4,5]. However, identifying potential patients who would benefit from a long-term prophylaxis regimen is imperative to decrease the morbidity and mortality associated with HAE.

Some of the major concerns associated with the new recently approved and soon to be approved prophylactic medications is not only expense, but also how the drug is administered. Currently, C1-inh is available only via IV administration and its administration by a health care provider at a health care facility would be time consuming and inconvenient for the patient. In order to regain flexibility and lead to an increased quality of life for the patient, it would be prudent to determine who would be a candidate for self-administration of C1-inh and other IV medications. This manuscript will also review when and for whom self-administration would be a feasible, safe and effective option for prophylaxis and on demand with C1-inh. This is especially important since early therapy reduces the burden of disease.

Up until recently, when a patient experienced an attack, the treatment has been supportive care, hydration, pain relief, and close observation. FFP has been utilized successfully, but a small amount of risk is possible [6]. In Oct 2009 human C1-inh concentrate given at a dose of 20 U/kg was found to be safe, well tolerated and efficacious in diminishing time to relief onset when giving during acute facial or abdominal HAE attacks [7]. This treatment, although it ameliorates symptoms rapidly, still has multiple disadvantages for the patient if used after the symptoms of an acute attack start and many recommend starting "on demand therapy (ODT)" at the time of prodromal symptoms to decrease morbidity and possible mortality associated with HAE [6,7]. We will expand on this concept of prodromal symptoms and their significance regarding treatment in the text of this manuscript.

## Methods

A literature review (utilizing PubMed, OVID and Google) was performed using the following terms to search for individuals who should be treated with long-term prophylaxis: "long term prophylaxis and hereditary angioedema." Additionally, the terms prodromal symptoms, hereditary angioedema and C1-inh were used to search for literature regarding the sensitivity and specificity of prodromal symptoms and their use in treating an imminent attack. Finally, PubMed and Google were used to search for individuals deemed fit to self-administer medications: "self-administration of C-1 esterase inhibitor and HAE."

## Results

### Who is a suitable candidate for long-term prophylaxis with C-1 esterase inhibitor?

The individuals deemed candidates for long-term prophylaxis were identified in a previous literature review and those situations are listed in appendix 1[5]. Additionally, patients who fail, have adverse reactions to or are unable to tolerate androgen therapy should be considered for prophylaxis with C1-inh.

Currently, the medications used for prophylaxis can include androgens, antifibrinolytics, and C-1esterase inhibitor. It is likely that the short half life associated with the bradykinin receptor antagonist (icatabant) and kallikrein inhibitor (ecallantide) will limit they use as prophylactic therapy. The androgen, danazol, is the current medication of choice for prophylaxis due to its cost effectiveness and ease of administration. However, danazol has numerous side effects that may lead to the discontinuation of the drug and/or noncompliance in some patients.

Danazol, a synthetic derivative of ethisterone, is effective in decreasing the severity and frequency of attacks in patients with HAE [4]. However, due to the numerous side effects, which include weight gain, virilization, menstrual irregularities, depression, headache and abnormal liver function tests, it is often poorly tolerated. In a long-term study of 118 patients with HAE, 30 (25.4%) patients had to discontinue the drug due to these adverse effects [4]. Not only does danazol often lead to the intolerable side effects noted above it has also been shown to have a negative effect on lipid profiles. This unfavorable lipid profile may also exist in the setting of increased blood pressure in some patients on long-term danazol therapy and a subsequent increased risk of cardiac and vascular disease [8]. Another frequent adverse event is the increase risk of liver disease, including hepatic cell necrosis, cholestasis and even to the development of hepatocellular adenoma and hepatocellular carcinomas. The adverse effects are dose related with increased dosages being associated with increased adverse effects [4,6,8]. Appendix 2 demonstrates the adverse effects of androgen therapy [6].

In addition to androgen therapy, other medications have been investigated as prophylactic agents for HAE. Icatibant, a specific antagonist of bradykinin B2 receptors, is currently approved in Europe for the treatment of acute HAE attacks. However, it is not suitable as a candidate for prophylaxis due to its relatively short half-life of 1.2-1.5 hours with SQ administration [9].

Ecallantide is an inhibitor of the protein kallikrein and as of Nov 2009 has been approved for the use of acute attacks of HAE in the United States. However, similarly to icatibant, its use as a prophylactic agent is limited, secondary to its short half-life, which approximates 2 hours [4].

Antifibrinolytics, such as epsilon-aminocaproic acid, has also been used as a prophylactic agent for HAE. It is used to inhibit the formation of plasmin and fragments of the Hageman factor, leading to the inhibition of kallikrein and bradykinin production [10]. Anti-fibrinolytics have been used not only in patients with HAE but also to control bleeding after cardiac surgeries and in other hematologic diseases. Its major side effects include hypotension, cardiac arrhythmias, rhabdomyolysis, and generation of thrombi and associated risk of emboli. Because of the side effect profile, limited effectiveness and need to dose frequently physicians have not utilized this therapy to the same extend as androgens [11].

In comparison to these agents, plasma derived nano-filtered-C-1 esterase inhibitor, known as Cinryze, has a half-life of 36-48 hours when administered intravenously and could lead to significant protection for 72 hours or greater [12]. However, due to its expense, the need for IV administration and need to re-dose every 3-4 days suggest it should be used in those with severe disease or in those that their HAE has a significant impact on their quality of life.

The use of nano-filtered-C-1 esterase inhibitor (nf-C1-INH) for prophylaxis has been well received in the USA. Dosing twice per week seems to be important to limit break through attacks, but even with twice weekly dosing acute attacks often occur requiring additional doses of nf-C1-INH. From personal communications with physicians prescribing nf-C1-INH most are encouraging patients to self-treat or be infused by family members. Some have advocated the use of indwelling central catheters or ports; however, the benefits of a port need to be weighted against the adverse events associated with them. In our cohort the use of nf-C1-INH infused through a port has been complicated with thrombi.

### Treating at the time of Prodromal Symptoms

Treatment at the time of prodromal symptoms, which may result in occasional over treatment, but which will still conserve concentrate and reduce cost when compared to prophylactic therapy, would decrease morbidity and mortality associated with HAE. Treatment before any swelling, onset of abdominal pain or throat swelling would improve quality of life of patients and reduce loss of productivity. Prior manuscripts by M Prematta and J Kemp, both published in 2009 in the Proceedings of Allergy and Asthma, demonstrated that prodromal symptoms are a sensitive predictor that an attack may occur in hours or days, but the exact specificity of prodromal symptoms for an attack is not known. The most common identifiable prodromal symptoms include unusual fatigue, rash on arms or legs and muscle aches. The retrospective study, noted above, conducted in 2009 by Prematta et al has already established that prodromal symptoms can be used as a sensitive measure to predict an acute attack [7].

**Figure 1:**
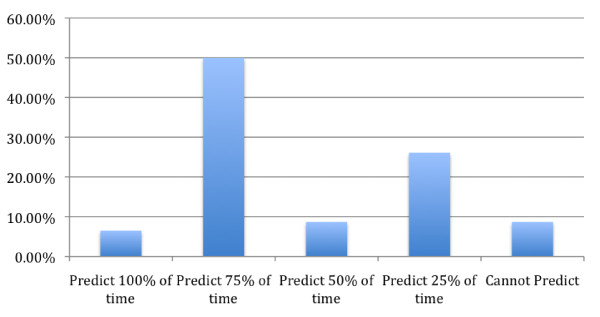
**Ability to predict HAE attacks based on prodromal symptoms**. This bar graph demonstrates the percentage of individuals with HAE who are able to predict an HAE attack based on prodromal symptoms based on the study by Prematta in 2009. 6.5% of patient are able to predict the onset of an attack 100% of the time, 50% are able to predict an attack 75% of the time, 8.7% are able to predict an attack 50% of the time and 26.1% are able to predict an attack 25% of the time. Only 8.7% are unable to predict their attacks at all.

**Figure 2:**
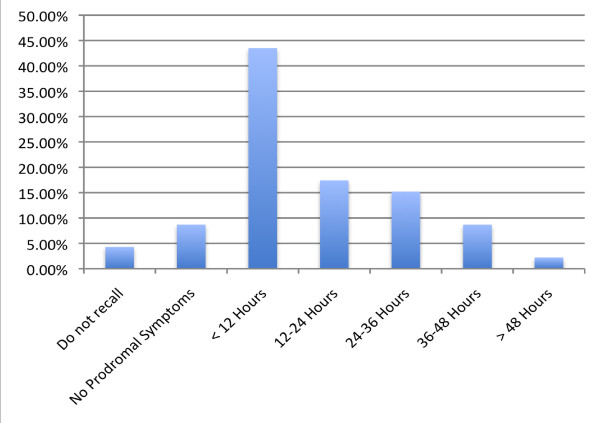
**Time between onset of prodromal symptoms and their next HAE attack**. This bar graph demonstrates the timing of acute attacks after the onset of prodromal symptoms. 45.5% of all patients had an attack within 24 hours of a prodromal symptom and 54.5% reported that their attack came 24 hours after the onset of the prodromal symptoms. However, the majority reported an attack within the first 12 hours after the onset of the prodromal symptom.

This study, utilizing a 4-page survey, was conducted in order to investigate the reliability with which prodromal syndromes can be used to identify an imminent attack. The results, demonstrated in figure 1 indicate that 3 (6.5%) patients could predict the onset of symptoms 100% of the time; 23 (50.0%) answered the ability to predict acute attacks 75% of the time; 4 (8.7%) patients answered 50% of time; and 12 (26.1%) answered 25% of the time. Only 4 (8.7%) reported not being able to predict the onset of HAE attacks [7]. Among the patients that remembered the timing of past prodromes, 20 of 44 (45.5%) patients reported that the average time of the onset of a prodrome was less than 24 hours before an HAE attack. Meanwhile, 24 of 44 (54.5%) patients reported that, on average, the onset of prodromal symptoms developed greater than 24 hours before HAE symptoms initiate. Figure 2 demonstrates these data [7].

These data support that prodromal symptoms occur commonly before acute HAE attacks with 87.0% of patients having had a prodrome before their last HAE attack, and 95.7% of patients reported having had a prodromal symptom before at least one acute attack in the past [7]. These data have demonstrated that prodromal symptoms could indeed be a sensitive measure of predicting acute HAE attacks and could possibly be used to initiate therapy before the onset of an acute attack, thus reducing morbidity and possibly mortality. In addition, this could lead to a better quality of life and decreased anxiety for patients with HAE [7].

### Who is fit to self-administer C1-inh at home?

The ability of patients to self-administer intravenous C1-inh at home would allow for greater flexibility, increased convenience and an increased quality of life, provided they were able to demonstrate the techniques noted in appendix 3 [13]. It also would decrease time to treatment if able to be administered by the patient for an acute attack, which should lead to a reduction of severity and duration of acute attacks. The benefit of self administration of prophylactic C1-inh would decrease cost and allow the patient significant flexibility to travel and administer therapy at the most suitable time. The current dosage recommended by the FDA for routine prophylaxis is 1000 units intravenously every 3-4 days and would require significant time and inconvenience to the patient if this had to be administered solely by a health professional.

Two studies have shown that select patients may benefit tremendously from self-administration of C1 esterase inhibitor and with self-treatment are able to improve quality of life [14]. These 9 patients were experiencing severe and frequent attacks of HAE. Their quality of life was assessed before and after 3 to 48 months of self-administered therapy. The QOL was assessed using the Dermatology Life Quality Index (DLQI) and the 36 Item Short form Survey questionnaires. The mean DLQI fell significantly from 12.6 +/- 4.65 to 2.7 +/- 1.38. The mean for the Short Form survey also improved significantly. No adverse events occurred during the 3-year period of intravenous self-administration [14].

In addition to affecting positively the patient's quality of life, one study also investigated whether self-administration was feasible and safe for the patients. Levi et al examined 31 patients using C1-inh as an "on demand" treatment and 12 patients using C1-inh prophylactically. Both groups were able to successfully administer the concentrate with a failure rate of less than 2%. During self administration attacks decreased from 4.0 to 0.3 per month in the group using prophylactic C1INH, but also self administration significantly decreased time to relief onset in those patients using it on demand for acute attacks [15]. This study not only confirmed the efficacy of self administered intravenous C1INH both as on demand treatment and as prophylactic therapy, but also demonstrated that patient administration is a viable and safe option. A manuscript published our manuscript further investigates self-infusion therapy and outlines the technique, quality assurance, training and reassessment of patients' prescribed self-infusion at home.

Unless peripheral access is limited, indwelling central catheters should be avoided due to the adverse events associated with port-o-catheters and similar devices. The most common complications of central lines include mechanical complications, infections and thrombotic events. Adverse events associated with indwelling central catheters are listed in appendix 4 [16].

Unfortunately, ecallantide has not been approved in the USA for home nor self-administration. The surveillance program required by the FDA for ecallantide limits its use to the clinic, and should be given by a health care provider who is capable of treating anaphylaxis, since anaphylaxis is a rare side effect of ecallantide. Dyax is hoping that the post-marketing surveillance program will demonstrate the safety of ecallantide allowing it to be self administered by the patient at home via the subcutaneous route.

Icatibant is presently repeating phase 3 studies in the USA and is anticipating approval for self-administration by the subcutaneous route. The drug is stable at room temperature and this combined with approval of icatibant for self subcutaneous injection will provide significant flexibility for patients with HAE to travel, camp, hike and do other recreational activities. It is projected that icatibant will be approved in the USA in 2012.

## Discussion

The treatment of acute attacks and prophylactic treatment of HAE has been evolving. In the recent past, the treatment of acute attacks was largely supportive, with hydration, pain relief and close observation as the mainstays of treatment. FFP has also been used, but does present increased risk for viral transmission as compared with C1-inh, and there have been anecdotal reports of exacerbations of an acute attack when FFP is given for treatment; however, it appears this is a very infrequent occurrence [6].

For prophylactic treatment, therapy has largely revolved around androgens, particularly danazol. However, as discussed previously, danazol has a negative side effect profile, which makes it intolerable for some patients. Other treatments, such as kallikrein inhibitors and bradykinin antagonists, are unlikely to be effective for prophylaxis due to their short half-lives. Antifibrinolytics are limited by their adverse effect profile.

Fortunately so nf-C1-inh is now available for use as prophylactic therapy. It is approved for 1000 U every 3-4 days, but due to breakthrough attacks, higher doses are being investigated to see if better control can be achieved. Even with breakthrough attacks, it appears that regular use of C1-inh reduces the severity and duration of the breakthrough attacks. This prophylactic regimen, although it has a less negative side effect profile than danazol, has a high cost and requires intravenous administration. Using a health professional for infusions can be quite time consuming, frustrating and inconvenient for the patient. The concept of self-administration has been also proven reasonable and effective, but would require correct patient selection and teaching.

Currently, on demand C1-inh (ODT) has also been proven safe and efficacious when used at the onset of a facial or abdominal attack. However, since it is used at the onset of an attack, multiple disadvantages for the patient still exist, such as pain and lost of work or school. C1-inh has been used successfully for 30 years in Europe as ODT for acute HAE attacks has been shown to be safe and effective and is the preferred therapy in Europe at this time [17].

For future therapy, the idea of ODT, would allow treatment based on prodromal symptoms experienced by the patients. As discussed in the text, up to 50% of individuals can predict 75% of their attacks based on prodromal symptoms. Although some selection bias may have been introduced in this study, as those who do have prodromal symptoms may have been more likely to respond, the data still demonstrate a significant portion of people who could benefit from ODT. These prodromal symptoms, be it fatigue, rash or muscle aches, are often followed by an attack, usually within hours to days. This would allow patients who do experience prodromal symptoms to preemptively treat themselves in the hopes that it would prevent an imminent attack and the symptoms that cause pain, disfigured appearance and even death. Although this method could lead to the occasional over treatment, it would hopefully lead to decreased morbidity and a better quality of life. The effectiveness of ecallantide and icatibant, both short acting therapies, for prodromal symptoms needs to be assessed, but we anticipate efficacy.

As evident from our results self-administration is a key feature for patients to treat and control their disease. Subcutaneous injection is obviously preferred over intravenous therapy as the technique is easily taught and the adverse events associated with poor technique are minimal. This is in contrast to intravenous therapy where guidelines are needed for teaching, assuring quality and infection prevention via continual evaluation, in addition to preventing other adverse outcomes that may occur with intravenous lines. Adverse events associated with indwelling central lines are far greater and more likely associated with significant morbidity and possible mortality and because of this should be avoided unless access peripherally is severely compromised (see appendix 4) [16].

Both acute and prophylactic treatment of HAE has been changing since the approval and introduction of C1-inh concentrate in the USA. Although currently approved for both acute and prophylactic treatment of HAE, the idea of ODT for use of prodromal symptoms may broaden the use of C1-inh. Currently, cost and its administration route are drawbacks of C1-inh, but many studies have already shown that self-administration is feasible and safe as long as proper candidates are selected. The multiple advances in prophylactic treatment and therapy for those suffering from HAE are exciting and may represent a better quality of life for those individuals suffering from repeated attacks. With the hopeful prospect of ODT for prodromal symptoms, HAE attacks may become more infrequent still and can help these individuals maintain control over their disease and lead an attack free life.

## Abbreviations

C1-inh: C1 esterase inhibitor; nf-C1-INH: nano-filtered-C-1 esterase inhibitor; HAE: hereditary angioedema; ODT: on demand therapy.

## Competing interests

TC discloses that he is a speaker for Dyax, Viropharma, CSL Behring and participates in research for Dyax, Viropharma, CSL Behring, Pharming and Shire. CD has nothing to disclose.

## Authors' contributions

TJC conceived and coordinated the study. CD carried out the research and drafted the manuscript. TJC edited the manuscript and both authors read and approved the final manuscript.

## Appendixes

### Appendix 1

Modified from Craig et al, Annals of Allergy asthma and Immunology, 2009 [5]

Candidates for long-term prophylaxis. Individuals who suffer from the listed consequences of their HAE and hence have a diminished quality of life are candidates for prophylaxis with C1-inh.

Those deemed candidates for long-term prophylaxis with C-1 Esterase Inhibitor.

-Those with significant anxiety

-Those with more than 1 attack per month

-Previous intubation or ICU stay

-Previous laryngeal swelling

-Those with more than 10 days lost from school or work per year

-A significantly decreased QOL

-Narcotic dependence

-Those with limited access to healthcare or with rapid onset of attacks

### Appendix 2

Modified from Craig et al, Proceeding of Allergy and Asthma, 2007

Adverse Effects of Danazol [6]

Adverse events associated with danazol. This appendix demonstrates the numerous adverse events associated with long-term administration of the attenuated androgen, danazol. These multiple side effects often lead to noncompliance or discontinuation of the drug.

-Weight gain

-Virilization

-Menstrual irregularities

-Depression

-Headache

-Abnormal LFTs

-Negative effect on lipid profiles

-Cardiac and vascular disease

-Liver disease including hepatic cell necrosis, cholestasis, hepatocellular adenoma and hepatocellular carcinoma

-The need to follow LFTs, lipid panels, and liver imaging

### Appendix 3

Adapted from Nentwich, Intravenous Therapy, 1990 [13]

Procedure for Self-Infusing of C-1 Esterase inhibitor

Procedure for self-administration of IV medications. The necessary procedure that must be demonstrated in order to be able to successfully self-administer IV medications is listed. Careful selection of the proper patient is essential in order to ensure compliance.

Patient must demonstrate the following technique

1. Cleanse skin with alcohol and betadine

2. Prepare medication in aseptic technique

3. Apply tourniquet

4. Insert butterfly

5. With blood **splash**, inject saline to keep line patent and remove tourniquet

6. Tape needle down

7. Infuse drug over 10-20 minutes

8. Complete all steps with aseptic techniques

9. Remove needle when complete

10. Apply pressure for a few minutes

11. Bandage area

### Appendix 4

Modified from McGee et al NEJM, 2003 [16]

Adverse events associated with indwelling central catheters. Indwelling catheters are associated with many significant adverse events, some which can be life threatening.

Adverse events associated with indwelling central catheters and other similar devices

-Arterial puncture

-Hematoma

-Pneumothorax**

-Infection

-Thrombosis

**Depends on site of insertion
